# Recent Advances in Conjugated Polymer-Based Biosensors for Virus Detection

**DOI:** 10.3390/bios13060586

**Published:** 2023-05-28

**Authors:** Thanh Ngoc Nguyen, Viet-Duc Phung, Vinh Van Tran

**Affiliations:** 1 NTT Hi-Tech Institute, Nguyen Tat Thanh University, 300A Nguyen Tat Thanh, Ward 13, District 4, Ho Chi Minh City 700000, Vietnam; nnthanh1080@gmail.com; 2 Institute of Fundamental and Applied Sciences, Duy Tan University, Ho Chi Minh City 700000, Vietnam; phungvietduc@duytan.edu.vn; 3 Faculty of Environmental and Chemical Engineering, Duy Tan University, Da Nang 550000, Vietnam; 4 Department of Mechanical Engineering, Gachon University, Seongnam 13120, Republic of Korea

**Keywords:** conjugated polymer, optical biosensors, conjugated polymer hydrogels, organic field-effect transistors (OFETs), organic electrochemical transistors (OECTs)

## Abstract

Nowadays, virus pandemics have become a major burden seriously affecting human health and social and economic development. Thus, the design and fabrication of effective and low-cost techniques for early and accurate virus detection have been given priority for prevention and control of such pandemics. Biosensors and bioelectronic devices have been demonstrated as promising technology to resolve the major drawbacks and problems of the current detection methods. Discovering and applying advanced materials have offered opportunities to develop and commercialize biosensor devices for effectively controlling pandemics. Along with various well-known materials such as gold and silver nanoparticles, carbon-based materials, metal oxide-based materials, and graphene, conjugated polymer (CPs) have become one of the most promising candidates for preparation and construction of excellent biosensors with high sensitivity and specificity to different virus analytes owing to their unique π orbital structure and chain conformation alterations, solution processability, and flexibility. Therefore, CP-based biosensors have been regarded as innovative technologies attracting great interest from the community for early diagnosis of COVID-19 as well as other virus pandemics. For providing precious scientific evidence of CP-based biosensor technologies in virus detection, this review aims to give a critical overview of the recent research related to use of CPs in fabrication of virus biosensors. We emphasize structures and interesting characteristics of different CPs and discuss the state-of-the-art applications of CP-based biosensors as well. In addition, different types of biosensors such as optical biosensors, organic thin film transistors (OTFT), and conjugated polymer hydrogels (CPHs) based on CPs are also summarized and presented.

## 1. Introduction

Viruses have been becoming a major threat to the wellbeing of humans, animals, and plants. In the past, there were several notable worldwide pandemics such as the 1918 influenza [1], human immunodeficiency virus/acquired immunodeficiency syndrome (HIV/AIDS) [2], 2009 swine-influenza A (H1N1) [3] causing millions of deaths. To date, the COVID-19 pandemic, an infectious disease that is caused by the severe acute respiratory syndrome coronavirus 2 (SARS-CoV-2), has remained and seriously affected the human health and the social and economic development [4]. In general, development and use of several approved vaccines along with the self-quarantine and good personal hygiene habits have been demonstrated as effective solutions to prevent and stop the fast spreading of viruses. Nonetheless, the development of effective, rapid, and low-cost techniques for early and accurate virus detection has been utterly important and should be given priority for prevention of such pandemics. To date, real-time reverse-transcription polymerase chain reaction (RT-PCR)-based techniques are still regarded as a gold-standard technology for detection of virus pandemics, especially SARS-CoV-2, in combination with other direct or indirect techniques, i.e., computerized tomography (CT) scans, enzyme-linked immunosorbent assays (ELISAs), and serological assays [5,6]. Although these methods have proven their own efficiency in virus detection, there have been still several drawbacks and problems significantly encountering their practical applications, as presented in Table 1. Such techniques often demand expensive equipment, expertise operators, time-consuming and labor-intensive work to acquire the desired results [7]. 

Biosensors and bioelectronic devices have appeared as a potential strategy to suppress the above issues of the current detection methods [8]. Furthermore, biosensor platforms have also been considered as the next-generation diagnostic technologies for COVID-19 and other pandemics owing to their superior advantages related to the sensing robustness, rapid detection, use of a minimum analyte concentration, and miniaturized devices [9,10,11]. Nanotechnology and advanced materials play a crucial role in the rapid advancement and development of biosensors through an increased sensitivity and specificity to virus analytes. Advances of novel materials in the design and fabrication of biosensors have created opportunities to develop and commercialize biosensor devices in the healthcare industry. Numerous materials, i.e., gold and silver nanoparticles, carbon-based materials, metal oxide-based materials, and graphene have been applied for preparation of high-performance biosensors [12,13]. Among them, conjugated polymer (CPs) is a promising candidate for fabricate excellent biosensors with high sensitivity and selectivity to viruses due to their outstanding features related to the unique π orbital structure and chain conformation alterations [14]. Therefore, the development of innovative technologies based on CP-based biosensors has been expected to attract huge attention among potential research related to early diagnosis COVID-19 as well as other virus pandemics.

It has been crucial to consolidate the existing scientific evidence for applicability of CP-based biosensor technologies in virus detection. Here, we provide a critical overview and evaluation of the existing works in the field of CP-based biosensors for identifying various viruses. Specifically, we briefly introduce and emphasize structures and interesting characteristics of different CPs for biosensor applications. Moreover, the state-of-the-art applications of CP-based biosensors in detection of viruses are summarized and described in detail. Different potential kinds of CP-based biosensors including optical biosensors, organic thin film transistors (OTFT), and conjugated polymer hydrogels (CPHs) are also discussed and presented. Therefore, this review provides an outlook on the future of CPs in biosensor devices and their potential for the development of point-of-care technologies (POCT) for early virus diagnosis.

## 2. Structures and Characteristics of Conjugated Polymers for Biosensors

CPs are defined as macromolecules generally consisting of a π-conjugated sp2 carbon-based backbone surrounded by aliphatic side chains, which induce the superior features such as intrinsic flexibility, optoelectronic properties, and solution processability [15,16]. CPs possess interesting properties related to a combination of electrical conductivity and characteristics of organic polymers. Moreover, owing to strong light-harvesting, good fluorescence quantum yield, and high photostability, CPs have been regarded as one of the most promising candidates for development of biosensors and imaging technologies [17]. π-CPs molecules contain alternating saturated and unsaturated bonds [18], and the signals including electrical charges or energy states can be fast migrated by this conjugated π-electron system. Moreover, the electrical charges or energy states also generate the high electrical conductivity for CPs [19], and the conjugated π-system can provide multiple CP monomers to facilitate interaction with biomolecule analytes, thereby resulting in the signal amplification effect [20]. CPs can be used for design of biosensor devices with two different roles, i.e., active components or passive supporting and structuring materials. In the role of passive supporting materials, CPs are used to support and immobilize the detecting biocomponents [21]. On the other hand, CPs are often employed and served as transducers (active components) in biosensors, which mediate the signal upon analyte interaction. Compared to biosensors using other molecules, therefore, the sensor devices based on CPs exhibited higher sensing performance [22]. For CP-based biosensors, CPs are possible for designing two main types of transducers: (i) electrical transducer which records electrical signals via changes in resistivity, in electrical current, or in potential [23,24]; and (ii) optical transducer receives optical signals which are manifested by changes in fluorescence wavelength, intensity, or colors [25,26,27].

Owing to the outstanding features, the CPs are considered as one of the most important materials in fabrication of both electrochemical and optical biosensors for virus detection [9,14,28]. There are two basic types of CPs that can be used to employ biosensors including conjugated homopolymers and copolymers. Conjugated homopolymers such as poly(acetylene), poly(3,4-ethylenedioxythiophene) (PEDOT), poly(thiophene), poly(p-phenylenevinylene) (PPV), poly (pyrrole) (PPy), and poly(aniline) (PANI) are defined as normal CPs consisting of single species of repeating units [29], whereas a conjugated copolymer is a CP containing two or more different types of repeating units (more than one type of monomer) such as DPP-DTT, PEDOT:PSS, and TBTT-ProDOT [27]. Compared to homopolymers, copolymers are special CPs with excellent optical luminescent and electrical conducting properties due to their extensive π-conjugation [30]. Moreover, copolymers also possess some advantages in development of advanced biosensor devices such as tailorable structures, tunable optoelectronic properties, processability, high solubility and stability, and ease of device fabrication [31]. The properties of common homo- and co-CPs used for preparation and design of biosensors are summarized in Table 2.

**Figure 1:**
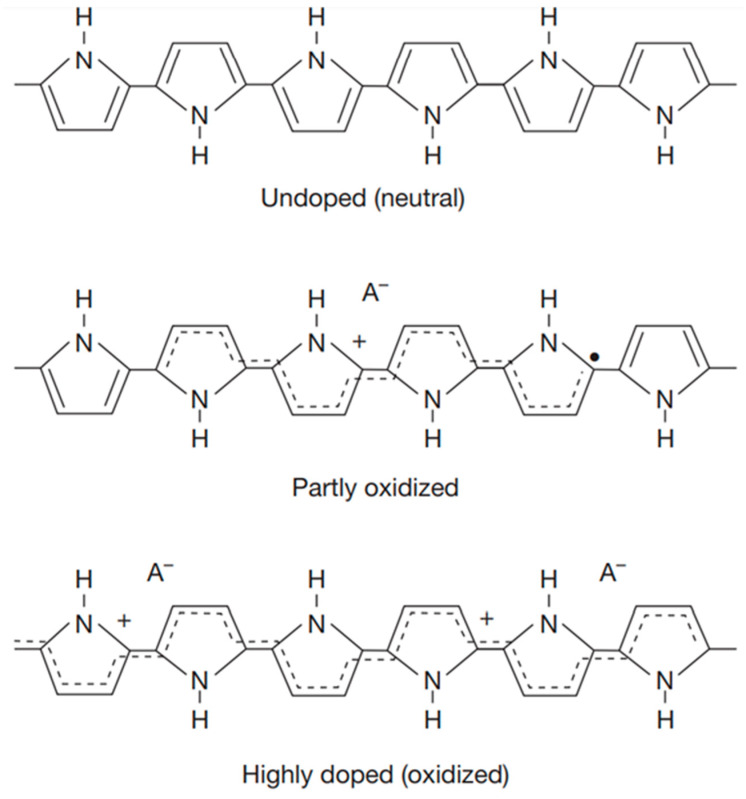
The PPy structures at different oxidation levels after the electrochemical or chemical doping [41].

CPs are basically prepared by two approaches including chemical and electrochemical methods [14,32]. Some desirable properties of CPs are enabled by tailoring and modulating their molecular weight, sequence, and end groups. Thus, controlled polymerization methods with high reproducibility during the synthesis and preparation process play a crucial role for producing novel CPs [33]. Electrochemical polymerization is often preferred to be used for preparation of CPs in biosensor applications due to several advantages including low temperature, a large surface area of microelectrodes, controllable thickness and shapes of CP films, and fast process (few seconds) [34]. Moreover, in the electrochemical polymerization method, the CPs are also deposited on the electrode surface by coupling reactions of oligomers produced by radical cations of oxidized monomers [35]. Recently, doping has been demonstrated as a promising method to increase in the hole mobility of CPs [36,37], thereby improving the sensitivity of CP-based biosensors [38]. The pristine state of CPs often exhibits low conductivity and mobility; thus, the doping process can significantly increase its electrical conductivity. The CP doping is able to be classified into four main approaches, including chemical doping, electrochemical doping, photochemical doping, and interfacial doping [39]. In chemical doping, the charge transfer redox is a main mechanism, and the CP doping can be conducted in two ways such as p-type doping or n-type doping. The p-type doping is accomplished by partially oxidizing the CP backbones using halogens, i.e., iodine, bromine, or chlorine (Figure 1) [40,41], whereas n-type doping can be achieved by reduction of CPs using a strong reduction potential such as sodium organic compounds [42]. For electrochemical doping, the principle is mainly the same as that of the chemical doping process but using an electrode to supply the redox potential. Therefore, the electrochemical doping of CPs is highly influenced by the potential and counter ions from the surrounding electrolyte solution. The electrochemical doping is the most commonly used doping technique for fabrication of CPs in biosensor applications, especially electrochemical biosensors [43]. Recently, most research has concentrated on studying and using the p-type doping process to improve the electrical conductivity of CPs [44].

**Table 2:** Summary of important properties of common conjugated polymers for development of CP-biosensors.

Conjugated Polymers	Conductivity (S/cm)	Properties	Refs
Homopolymers			
Polyacetylene	Trans-polyacetylene (4.4 × 10^−5^ ) Cis-polyacetylene (1.7 × 10^−9^)	Water insolubility Low solubility in organic solvents The morphology consists of fibrils, with an average width of 200 Å	[45,46]
Polypyrrole (PPy)	2–100	Solubility in DMSO, chloroform, chlorobenzene, and tetrachloromethane Environmental stability, compatibility	[47]
Polythiophene (PT)	10–103	Insolubility in ordinary solvents The optical properties of PTs are sensitive to many factors. PTs exhibit absorption shifts due to application of electric potentials	[48]
Poly(phenylenevinylene) (PPV)	10–13	Water insolubility A highly ordered crystalline thin film. A small optical band gap and bright yellow fluorescence	[49]
Poly(aniline) (PANI)	30–200	Insolubility in the common organic solvents Solubility in NMP, DMSO, DMF, and THF Environmental stability and compatibility	[50]
Poly(3,4-ethylenedioxythiophene) (PEDOT)	0.4–400	Available aqueous dispersion Low density, excellent thermoelectric performance	[51,52]
Polydiacetylene (PDA)	∼10^−5^	Highly ordered backbones with customizable side chains An absorption peak at ∼640 nm (blue color). Upon interaction with external stimuli, the main absorption peak shifts hypsochromically to ∼540 nm (red color)	[53]
Copolymers			
DPP-DTT	8.4	Solubility in chloroform, chlorobenzene, and dichlorobenzene High mobility 10 cm^2^/Vs	[54,55]
PEDOT:PSS	54	Self-healing properties Adjustable conductivity, good transparency to visible light, excellent thermal stability, and a high level of biocompatibility High water solubility	[56,57]
TBTT-ProDOT	-	Optical bandgaps ranging from 1.96–2.46 eV High photoluminescent quantum yields	[31,58]

## 3. CPs-Based Optical Biosensors for Virus Detection

CPs have attracted huge attention as effective optical transducers in optical biosensor for virus detection due to their excellent light absorption and emission properties. Particularly, water-soluble CPs such as poly(fluorene phenylene), polyfluorene, and polythiophene have been recently employed as optical probes for developing and designing fluorescent and colorimetric biosensors.

### 3.1. Fluorescent Conjugated Polymer Biosensors

**Figure 2:**
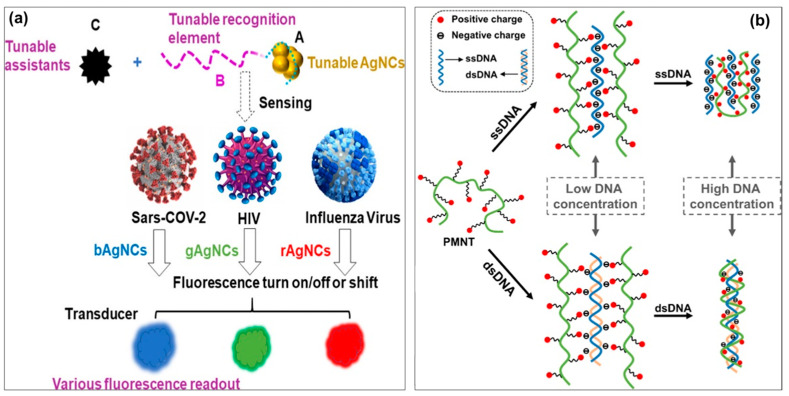
(**a**) Schematic illustration of the working mechanism of CP-biosensors in detection of various viruses based on different fluorescent readouts. Different types of CPs indicate blue, green, and red fluorescents, respectively. The various readouts (filled with blue, green, and red colors) indicate the sensing signals are obtained from different fluorescent CPs [59]; (**b**) Proposed scheme representing the binding mechanism between PMNT and ssDNA/dsDNA [61].

Fluorescence analysis has been considered as one of the most promising technologies in biosensing applications due to its superior advantages such as high sensitivity, simple operation, and strong specificity. Recently, DNA-CPs have been used as effectively fluorescent probes for detecting various viruses. The CP-based fluorescent biosensor is presented in Figure 2a, which mainly includes two domains A and B [59]: A domain is a CP template, and B domain is a specific probe to a targeted virus. Moreover, a C factor can be additionally introduced to enhance the fluorescent interaction. For the detection mechanisms of CP-based fluorescent biosensors, the fluorescence of CPs may be turned on/off or shift upon the virus presence [60]. The fluorescent biosensor based on DNA-CPs can generate different fluorescence readouts by charge attraction, thereby leading to detection of different viruses via their DNA sequence [59]. For instance, Zhang et al. successfully fabricated a label-free fluorescent CP-based DNA biosensor using a cationic CP, poly(3-(3′-N,N,N-triethylamino-1′-propyloxy)-4-methyl-2,5-thiophene) (PMNT) (Figure 2b) [61]. This study demonstrated that the fluorescence of PMNT polymer showed a significant change at 530 nm and a peak at 580 nm upon interacting with single-stranded DNA (ssDNA). The PMNT CP interacted with DNA molecules by electrostatic interactions and DNA base-mediated interactions. The results indicated that the PMNT-based fluorescent biosensor exhibited a high sensing performance with a detection limit of 1 nM DNA.

Currently, there have been some detection modes of CP-based fluorescent biosensors including (i) fluorescent enhancement (turn-on); (ii) fluorescent quenching (turn-off); (iii) fluorescent color change. For the fluorescent enhancement mode, the presence of target analytes will produce bindings perturbing the electron density along the CP backbone or changing the conformation of the polymer chain, leading to a significant increase in CP fluorescence. The CP fluorescence is partly or completely quenched and turned off by non-radiative relaxation pathways in a chain or the intermolecular aggregation of CP chains in the fluorescent quenching mode [62,63]. The fluorescent color change is likely to gradually increase along with a change in electron density inside the CP backbone, and the driving forces causing this change are induced by polymer aggregation [64], conformational change [65,66], and electron energy transfer [26,67]. In the CP-based fluorescent biosensors, hybridization of a complementary sequence with the DNA/RNA viruses results in a considerable enhancement of the fluorescence intensity as well as color change because of changes in the DNA/RNA structure. Therefore, these mechanisms are exploited to develop and design biosensors for effectively detecting various viruses through their DNA or RNA. Furthermore, fluorescence resonance energy transfer (FRET) has been also used to amplify the fluorescent signal in the CP-based fluorescent biosensors [68,69]. Regarding this approach, the energy of CPs is transferred to a fluorophore or quencher relying on an energy transfer mechanism between two chromophores [70]. The fluorescent color change mode is triggered depending on the changed conjugation length of a CP backbone, thereby leading to the shifted wavelengths of the absorbed light by the CPs.

Fluorescent biosensor devices for virus detection transduce biological recognition events into measurable signals in the presence of viruses. In general, water-soluble CPs have been widely used for design of solution-state biosensors, and these sensor devices work based on three types of signal transduction mechanisms: electron transfer, FRET, and analyte-induced aggregation of CPs [71]. Compared with other inorganic or small molecule counterparts, CPs enable the trapping of energy and/or migrating electrons along the conjugated backbone due to their good light-harvesting abilities [72]. For the electron transfer mechanism, the CP-based biosensor devices can induce a superquenching fluorescent effect by the appearance of quenchers through an effective electron transfer process. In this sensing system, the quenchers are often used as energy acceptors to allow transferring energy from the CP to the acceptor via FRET [26]. It has been demonstrated that FRET was developed based on the Forster theory which is generated from dipole–dipole interactions [71]. For the aggregation mechanism, the presence of virus analytes can induce the π-stacking phenomenon of the CP backbones, and the water-soluble CPs are aggregated by interchain interactions, which results in the fluorescence quenching.

### 3.2. Colorimetric-Conjugated Polymer-Based Biosensors

Due to the high ability in the amplification of sensory signals, CP-based fluorescent biosensors are considered as an ideal technique for detection of extremely small quantities of virus analytes. However, they require high-tech tools and expensive spectrophotometer instruments. Therefore, colorimetric biosensors are currently regarded as the most convenient and effective approach without using equipment for quickly detecting various viruses [73,74,75]. Colorimetric biosensors can be employed for identifying and quantitatively measuring virus analytes through color changes which can possibly be recognized by the naked eye or with simple portable optical detectors. Thus, colorimetric biosensors have attracted huge attention in the development of optical biosensors due to their high accessibility, cheap costs, simplicity, practicality, and high sensitivity and selectivity [76]. In general, colorimetric biosensors, defined as a special type of optical sensors, change or switch the color by a change in the environmental properties (external stimuli) such as physical or chemical environments. Recently, CPs have gained great interest as an excellent candidate for design and preparation of colorimetric biosensors due to their high sensitivity with significant changes in physicochemical properties [77,78]. Moreover, CPs are likely to be used in the rapid detection and biorecognition of elements of viruses, such as antibodies and proteins, because of the instantaneous transduction of stimuli into response [79]. Among many CPs employed for fabrication of colorimetric biosensors, PANI, polythiophenes, and PDAs are the most common CPs used for detection of pathogens and viruses [18,79,80]. Based on the designs and architectures, CP-based colorimetric biosensors can be divided into two basic types: solution-based CP biosensors (non-substrate) and substrate-based CP biosensors.

#### 3.2.1. Solution-Based CP Colorimetric Biosensors

**Figure 3:**
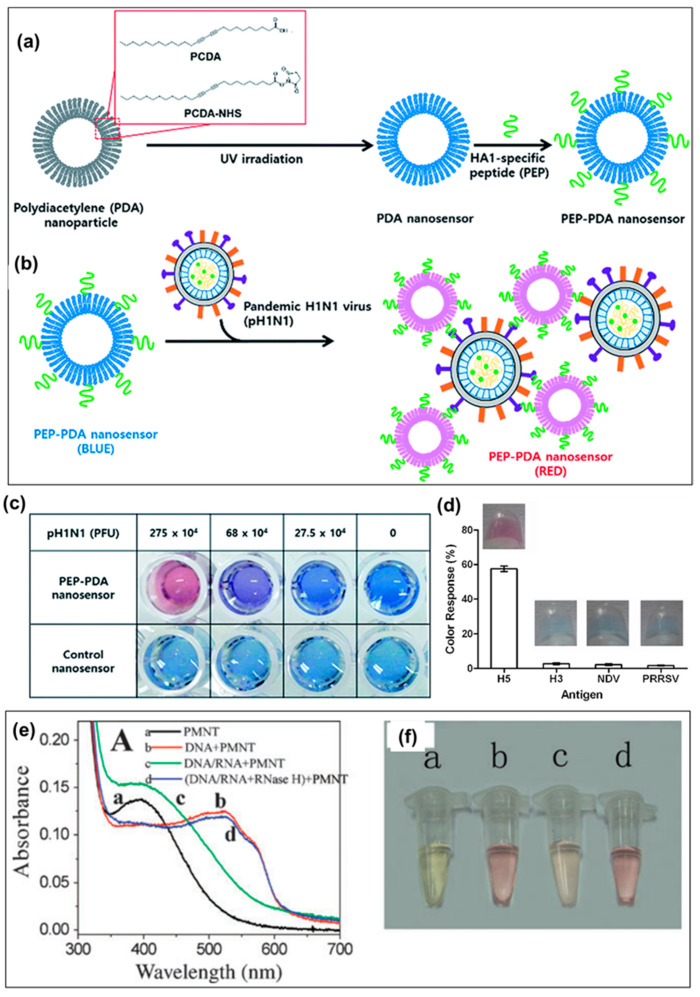
(**a**,**b**) Schematic illustration of the peptide-functionalized polydiacetylene (PEP-PDA) colorimetric biosensor for pH1N1 detection; (**c**) Colorimetric response of the PEP-PDA biosensor to different concentrations of pH1N1 [80]; (**d**) Colorimetric response of the PDA vesicle-based biosensor to different pathogens: H5—H5N1 influenza virus, H3—H3N2 influenza virus strain, NDV—Newcastle disease virus LaSota strain, and PRRSV—porcine reproductive and respiratory syndrome virus VR2332 strain [86]. (**e**) UV–vis absorption spectra and (**f**) optical images of PMNT with various target molecules: (a) pristine PMNT; (b) DNA; (c) DNA/miRNA; and (d) DNA/miRNA + RNase H [94].

The motivation for creating and developing aqueous solution-based CP colorimetric biosensors has originated from the state of clinically interesting target molecules and outstanding features of CPs. In biosensors, biological targets (i.e., DNA/RNA, proteins, or antibodies) often need to be dissolved and maintained in an aqueous environment. Furthermore, some CPs are highly water-soluble, which are easily dissolved and interacted with aqueous solution of target molecules. Many solution-state colorimetric biosensors using CPs have been fabricated for the detection of viruses. Among them, the PDA polymer is often used as a colorimetric sensing material in the fabrication of solution-state colorimetric biosensors due to its unique chromatic properties [81,82]. Most PDA colorimetric biosensors are prepared by incorporating specific bio-receptors into the polymer matrix or liposome. The PDA backbone conformation is changed by binds between target molecules and receptors, leading to the changed color from blue to red [83]. For instance, Song et al. developed the PDA colorimetric nanosensor by functionalizing PDA with a peptide (PEP) for identifying the pandemic H1N1 virus (Figure 3) [80]. This biosensor was prepared by simultaneous combination of PDA nanoprecipitation and modification with PEP to produce a specific and selective recognition to the pH1N1 virus. Specifically, 10,12-pentacosadiynoic acid (PCDA) and PCDA-NHS monomers were self-assembled and precipitated to obtain PDA nanoparticles in the aqueous phase, followed by UV irradiation to form the blue-colored PDA biosensor (Figure 3a). Hemagglutinin (HA) proteins play a vital role in aiding the virus to first adhere to and to enter a host cell, and thus, they are often used as bioreceptors to recognize specifically viruses, especially influenza virus [84]. The PEP-PDA biosensor was completely obtained by decoration of HA1 protein receptors through binding with PEP-modified PDA molecules, and this colorimetric biosensor could accurately recognize pH1N1 viruses because of a high affinity of HA1 receptors with the H1 strain (Figure 3b). Importantly, the PDA colorimetric biosensors have been demonstrated to be capable of detecting H1N1 virus by only the naked eye due to the unique chromic characteristics of PDA molecules. The PEP-PDA biosensor showed a colorimetric response with an obvious blue-to-red color change when reacting with pH1N1 viruses (Figure 3c), which is clearly visible to the naked eye. It has been indicated that the colorimetric transition of the PEP-PDA biosensor from blue to red can be explained by the perturbation of the conjugated ene–yne backbone of PDA cause by steric repulsion at the biosensor surface from the interaction of H1 strain and PEP [85]. This study demonstrated that PDA can be employed for fabrication and development of a commercially available kit based on colorimetric biosensors for virus detection.

Antibodies (immunoglobulins) are also known as one of the best receptors that can be employed to fabricate biosensors for detecting viruses [35]. Jiang and coworkers designed a highly sensitive, specific, and rapid solution-state biosensor based on PDA vesicles for detecting H5 influenza virus using the anti-H5 influenza antibody [86]. The PDA vesicles were formed by a combination of PCDA and 1,2-dimyristoyl-sn-glycero-3-phosphocholine (DMPC), followed by UV (254nm) irradiation. These PDA vesicles were integrated with bioreceptors (H5-mAb, monoclonal antibodies against the HA of H5 influenza virus) by covalent bonds and an EDC/NHS coupling reaction, while the excess carboxylic acid groups on the PDA versicle surface were blocked by bovine serum albumin (BSA) for prevention of non-specific adsorption. Such solution PDA vesicle biosensor showed a high performance in detecting H5 influenza virus: (i) a low LOD value (0.53 copies µL^−1^) and (ii) an obvious changed color from blue to red that enabled it to be recognized by the naked eye or simply measured by a UV-vis spectrometer. Furthermore, the colorimetric PDA biosensor exhibited high selectivity to H5 influenza virus in the presence of various pathogens including H3 influenza virus, NVD, and PRRSV (Figure 3d). In addition, this PDA biosensor also presented good results in detecting virus isolated from tracheal swabs in comparison with the RT-PCR technique. Except for influenza virus, solution-based PDA colorimetric biosensors have been demonstrated to be available for detection of other common viruses such as vaccinia virus infects [87,88], foot-and-mouth disease virus [89], bovine viral diarrhea virus [90], and hepatitis B [91]. These studies prove the possibility and potential of PDA in the development of solution-based colorimetric biosensors in effective detection of various viruses.

Polythiophenes and derivatives have also been employed for fabrication of solution-based colorimetric biosensors in detection of virus biorecognitions such as RNA because of their high water-solubility and structures [92]. Currently, detection of RNA viruses has still remained a big challenge due to the small size of miRNA, low annealing temperature, as well as the risk of cross hybridization. The length of the polythiophene derivatives containing 20–40 thiophene repeat units is demonstrated to match well with the miRNA virus size to generate the conformational changes [93]. Therefore, several studies have taken advantage of polythiophene derivatives for the development of simple, solution colorimetric biosensors to detect miRNA viruses without chemical labels and expensive instrumentation. For example, Zhang et al. developed an aqueous colorimetric biosensor based on a cationic polythiophene derivative (poly[3-(30-N,N,N-triethylamino-1-propyloxy)-4-methyl-2,5-thiophene hydrochloride (PMNT)) as an optical probe for detection of microRNAs from viruses [94]. In the solution, PMNT exists in a random-coil conformation, and its twisted conjugated backbone causes a decreased effect in the conjugation length, thereby resulting in an absorption peak at 400 nm and a yellow color (Figure 3e,f). Upon addition of a ssDNA receptor, the PMNT solution shifted to a red color and a new absorption peak at 525 nm derived from highly conjugated, planar conformation of PMNT. In the presence of a target miRNA, the triplex structure of the ssDNA/miRNA hybrid and PMNT molecules was formed, and PMNT in the triplex was less conjugated and a nonplanar conformation, leading to the same absorption peak of the triplex with PMNT itself but with greater absorption and an orange color. Interestingly, the authors demonstrated that this solution PMNT colorimetric biosensor was likely to be reproduced by digestion of miRNA using RNase H. Moreover, the PMNT biosensor could be carried out by simple spectrophotometers or even by the naked eye, considerably reducing the cost and increasing the test throughput. With a similar approach, Ho and Leclerc also developed an inexpensive solution colorimetric biosensor using a cationic polythiophene derivative for rapid detection and high-throughput screening of various target pathogenic proteins in the femtomole range [95]. In this study, ssDNA aptamer was first bonded to a cationic polythiophene through charge–charge interactions to obtain a solution colorimetric biosensor. The addition of pathogen proteins caused a polymer conformational change, and the biosensor worked by a color shift from yellow to red. More recently, Ammanath and coworkers successfully designed a flow-through colorimetric biosensor based on a cationic poly (3-alkoxy-4-methylthiophene) for detection of microRNA (mir21) and hepatitis B virus DNA (HBV-DNA) in plasma without requiring tedious sample pre-treatment and clean up protocols [96]. The biosensor could show colorimetric responses at nanomolar concentrations of mir21 and HBV-DNA from 1 nM to 10 mM, with a LOD of ~2 nM in plasma. The colorimetric biosensor system could be developed as a point of care disease diagnosis and used for detecting numerous virus biomarkers in plasma. The results from these studies demonstrated that polythiophenes and derivatives are potential candidates for the development of colorimetric biosensors that can be utilized to recognize virus biomarkers conveniently and rapidly.

#### 3.2.2. Substrate-Based CP Colorimetric Biosensors

**Figure 4:**
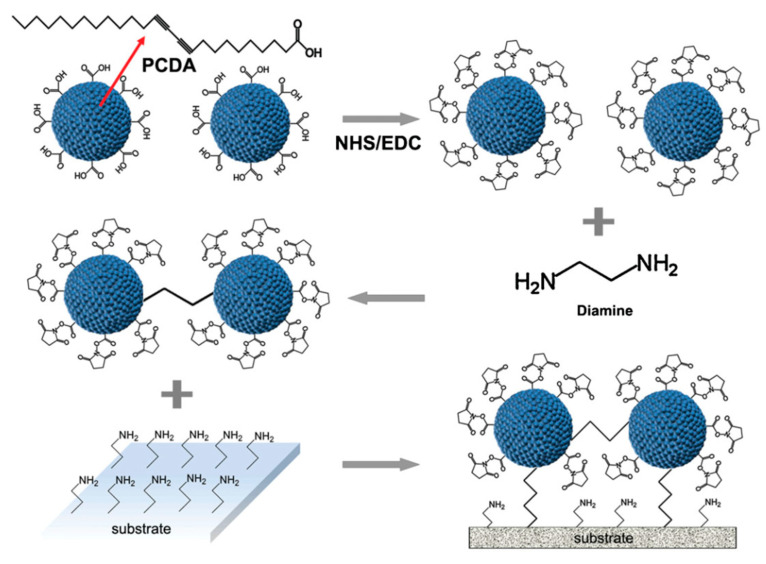
Schematic illustration of the preparation process of a substrate PDA biosensor based on crosslinking PDA liposomes and diamine for detection of pathogens [99].

Liquid-phase CP colorimetric biosensors are simply designed by dispersing CP molecules in a buffer solution, while solid-phase biosensors are prepared by immobilizing CP supramolecules onto substrate supports, facilitating the development of POCT devices with simplicity, flexibility, and portability. Although the solution CP colorimetric biosensors are convenient and low cost, their relative instability in solution has limited their applicability in practice. Hence, solid-phase colorimetric biosensors constructed on a substrate are ideal for the design of advanced colorimetric biosensors with simple, rapid, and robust platforms. For instance, a PDA solid colorimetric biosensor was reported for rapid identification of the influenza A (H1N1) virus by coating and immobilizing PDA nanovesicles onto a polyvinylidene fluoride (PVDF) substrate [97]. In this study, PDA nanovesicles were first prepared by functionalizing PDA with M149 antibody, and immobilization of these PDA nanovesicles on PVDF membrane showed a further improved stability and sample handling. However, currently conventional approaches for fabrication of solid-phase CP colorimetric biosensors have often relied on Langmuir–Blodgett (LB) or Langmuir–Schaeffer (LS) films [98]. These methods presented weak optical signals and required stringent conditions. On the other hand, solution colorimetric biosensors could show a stronger optical response due to use of CP supramolecules in a high concentration. Therefore, a potential strategy has been recently developed by integration and immobilization of liquid-phase CP biosensors onto a solid substrate, thereby effectively taking advantages of a liquid-phase CP dispersion for improving the sensing performance of a substrate colorimetric biosensor. Take, for example, Park et al. who developed a direct, multiplex colorimetric biosensor platform for identifying pathogens using a novel procedure to immobilize PDA liposomes (Figure 4) [99]. It has demonstrated that the instable immobilization of PDA liposomes on the solid substrate has been considered as a critical drawback for the design of the PDA liposome-based colorimetric biosensors. The study provided a promising strategy to overcome this problem by introducing an interlinker (ethylenediamine) which effectively works as a crosslinker to aid the immobilization of PDA liposomes. Using this strategy, the authors also successfully prepared a PDA colorimetric chip with the improved signal intensity and the high sensitivity. Furthermore, the substrate-based PDA colorimetric biosensor enabled it to perform the simultaneous quantitative and qualitative measurement of six different species of pathogens.

**Figure 5:**
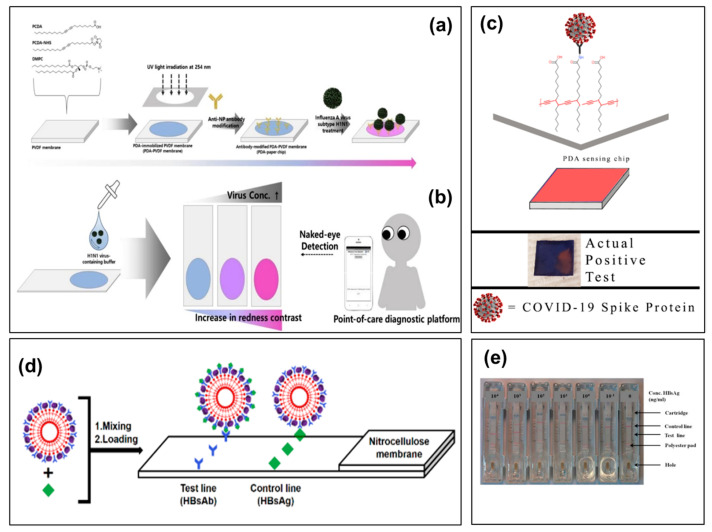
(**a**) Preparation process of PDA-paper chips and (**b**) the POCT system integrating PDA-paper chips for colorimetric detection of pH1N1 virus [103]; (**c**) PDA-paper biosensor for detection of the SARS-CoV-2 spike protein in artificial saliva [104]; (**d**) schematic illustration of preparation approach PDA nanovesicle-based colorimetric biosensor on nitrocellulose membrane and (**e**) its commercial kits [91].

In the last decade, development of colorimetric biosensors based on paper substrates has attracted large attention owing to several superior advantages including simplicity, rapidity, and low cost [100]. CPs are recently considered as an excellent candidate for the design of paper-based colorimetric biosensors because of their solution processability [101,102]. PDA is also a commonly used CP for fabrication of paper-based colorimetric biosensor devices that can serve as POCT platforms for detection of viruses. Son and coworkers recently introduced a PDA-based colorimetric biosensor for POCT detecting the influenza A virus [103]. This PDA-paper chip was prepared by immobilizing PDA molecules on the PVDF flexible substrate through a photo-polymerizing process, followed by decorating antibody bioreceptors into this PDA-PVDF membrane (Figure 5a). The PDA-paper colorimetric biosensor was demonstrated to show unique chromatic features related to a clearly changed color from blue to red in the presence of pH1N1 virus under different temperature and pH conditions. Especially, the PDA-paper chips were successfully integrated in a POCT system with special features: (i) visually recognizing viruses at a high concentration by the naked eye and (ii) identifying viruses at low concentrations using paper chips in combination with a developed program (App.) (Figure 5b). With a similar approach, Prainito et al. also developed a colorimetric, paper-based PDA biosensor for early detection of the SARS-CoV-2 virus via its spike protein in artificial saliva [104]. For maximizing the sensing performance, this PDA biosensor platform was fabricated with an optimal PCDA-NHS molar concentration of 10% via an esterification reaction of NHS and PCDA monomers. The SARS-CoV-2 spike proteins in saliva samples were used as biomarkers that induced a color change from blue to red when binding to the antibody receptors (Figure 5c). The paper-based PDA sensing platform showed a high sensing performance in detection of the SARS-CoV-2 spike protein, i.e., rapid and high responsivity within the concentration range of 1 to 100 ng/mL. Furthermore, pH and temperature conditions had little influence on the sensing ability of the paper-based colorimetric sensor. A smartphone image could be used to record and assess the sensing signal. These results demonstrated that PDA and CPs have been considered as potential materials for designing rapid, colorimetric paper-based biosensors to detect various emerging viruses, especially COVID-19.

Hepatitis B is an infectious disease, known as one of the 10 top leading causes of death in the world, which is caused by the Hepatitis B virus (HBV) [105]. Thus, the development of early diagnosis techniques for accurately detecting HBV virus has gained great attention among the general public and the scientific community. In recent years, the development of colorimetric biosensors based on the immunochromatographic assay (ICA) has been extensively investigated as a potential approach for early diagnosis of hepatitis B due to the rapid and straightforward detection of ICA [106]. Roh and coworkers developed a PDA commercial kit to selectively detect trace hepatitis B antigen by using PDA vesicles as a colorimetric indicator as well as a surface for immobilized hepatitis B surface antibody (HBsAb) [91]. First, the PDA/HBsAb complexes with sizes of ~200 nm were obtained by conjugation of HBsAb antibody and PDA through covalent linkage, followed by attaching these complexes on nitrocellulose membrane (Figure 5d). The PDA/HBsAb biosensor enabled to show the color change when target antigens interacted with antibodies, and the LOD was reported to be 0.1 ng/mL. In addition, the PDA/HBsAb complexes were also embedded on a commercial kit by lyophilization method, in which the complexes were loaded on the polyester pad and dried at 35 °C (Figure 5e). The colorimetric sensor kit was demonstrated to effectively detect up to 1 ng/mL HBV by the naked eye. Moreover, the PDA colorimetric kit also showed the superior advantages in terms of short inspection time, rapid reading time, easy handling, and economic feasibility. Therefore, the development of commercial kits based on PDA and other CPs will be a potential strategy.

## 4. CP-Based Organic Thin Film Transistors

**Figure 6:**
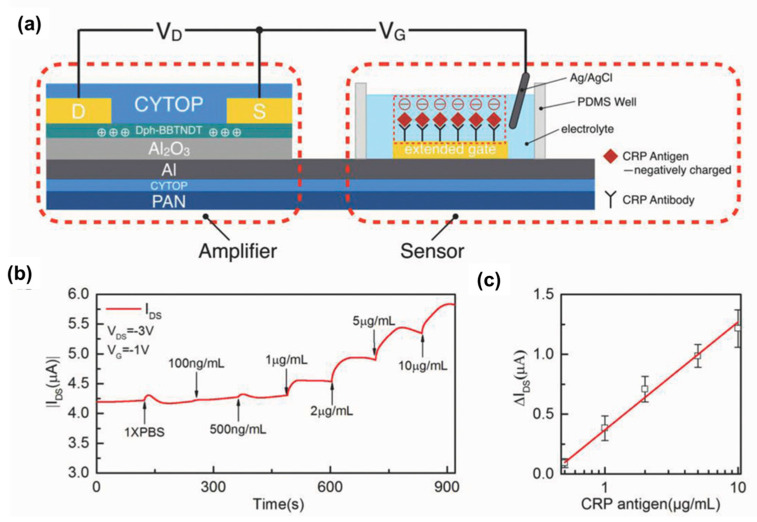
(**a**) Structure of novel OFET-based CRP sensor with an extended-gate electrode; (**b**) Increase of channel current and (**c**) calibration curve of increased channel current against CRP concentration [117].

Among numerous diagnostic methods, organic thin film transistors (OTFTs) have recently been considered as excellent candidates for the design of sensing devices due to simplicity in the fabrication process, inherent flexibility, light weight, biocompatibility, high sensitivity, low cost, and rapid response and instantaneous measurements [107,108,109]. The OTFTs are generally composed of three main functional components including a source, a gate, and a drain, in which the conducting channel between source and drain is controlled and modulated by an applied electric field at the gate [110]. For sensing applications, the OTFT translates input voltage signals from the gate electrodes into output signals (i.e., currents or voltages) at the drain electrode [111]. In the last decades, OTFTs-based biosensors have been extensively investigated and applied as innovative biosensing platforms because of their inherent capacity in transferring and amplifying biological signals into electrical signals. Among them, there are two main types of OTFTs including organic field-effect transistors (OFETs) and organic electrochemical transistors (OECTs) commonly used for virus detection [112]. OECTs have a similar structure and function to that of an OFET but employ an electrolyte between the channel and gate instead of a conventional dielectric with dipoles [113,114]. Recently, development of advanced materials with extraordinary properties, including high electronic conductivity, high carrier mobility, and large specific area, have been one of the key strategies for applications of OFET biosensors in virus detection [115]. CP semiconductors have been considered as promising materials for fabrication of high performance OFETs due to good solution processability, film-forming properties, and high flexibility. Among various CP types, p-type CP semiconductors, such as polythiophenes, PEDOT, PANI, and DPP-DTT, are often used for preparation of OFET biosensor devices due to their high efficiency and interaction with bioreceptors. For instance, Kergoat et al. reported a DNA sensor based on a water-gated OFET and nanostructured P3HT as an active channel [116]. In this sensor, DNA probes were immobilized onto the P3HT by covalent bonds. The OFET device showed a clear difference in the output currents upon DNA immobilization and hybridization. In this study, deionized water was utilized to increase the Debye length up to several hundreds of nanometers and decrease the off current upon DNA hybridization. Moreover, this device could operate at very low voltages (below 1 V) due to the formed electric double layer. However, CP-based OFET biosensors have remained a big challenge related to the degradation of CPs following exposure to moisture. Therefore, OFET biosensors are often designed with an extended-gate to separate the gate electrode (active channel) from the driving unit of OFET in order to overcome the above problem. For the “extended-gate”, a part of the gate electrode area was separately moved far from the active channel of the OFET. For instance, Ji and coworkers designed a sensitive OFET biosensor with a gold extended-gate for detection of C-reactive proteins (CRP) [117]. For the design of this device, the extended-gate was functionalized with CRP antibody and directly linked with a traditional Al gate (Figure 6a). In addition, for employing the biosensor configuration, another Ag/AgCl gate was connected with the extended Au gate via an electrolyte solution (PBS buffer), and thus, the extended-gate was able to be named as a floating gate. Based on the surface potential during antibody–protein antigen conjugation, this extended-gate could effectively control the net gate bias of the OFET device. It has demonstrated that the electrolyte-gated and extended-gate OFET significantly improved the sensing performance due to the modified capacitance and shifted the threshold voltage of the surface potential on the extended-gate, and the Debye’s screening length parameter minimized its influence in this type of OFET sensor. This OFET biosensor showed high responsivity and sensitivity with a wide range of CRP concentration (100 ng mL^−1^–10 μg mL^−1^) and LOD around 1 μg mL^−1^ (Figure 6b,c).

**Figure 7:**
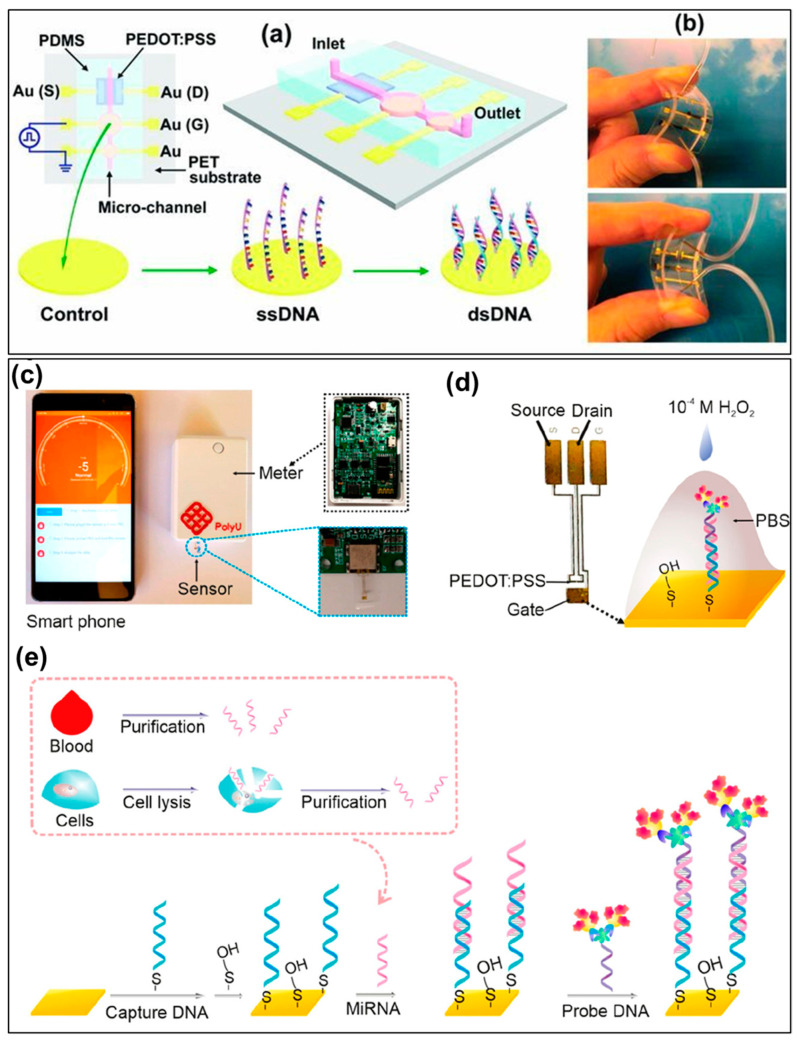
(**a**) Schematic illustration of an OECT embedded in a flexible microfluidic system for DNA detection; (**b**) an optimal image of the flexible sensor device [119]; (**c**) scheme of the OECT-based portable system connected with a smartphone via Bluetooth; (**d**) photo of an OECT miRNA sensor with three electrodes: drain, source, and gate; (**e**) scheme of a gate modification process [120].

OECTs have recently gained great interest for the design and development of highly sensitive biosensors in virus detection. More importantly, OECTs can be fabricated and integrated on flexible substrates, which is essential for the design of flexible sensor devices that can be attached on the human body [113]. For example, an OECT biosensor based on a PEDOT:PSS conductive channel was developed for effective detection of the human influenza A virus in aqueous conditions [118]. In this sensor system, an electrochemical amplifier of the OECT was utilized to provide the highly sensitive, selective, and label-free sensing performance to influenza A virus antigens. The changed drain current of the OECT happened after the virus adsorption onto the active channel, and the signal transduction mechanism was proposed by doping effect. The results indicated that the PEDOT:PSS-based OECT biosensor proved its higher efficiency in detection of influenza virus, compared with commercial immunochromatographic assays in the same detection time. Moreover, the OECT device was possibly integrated in the wearable system for monitoring the influenza virus owing to its printing processability as well as low power consumption. In another study, Lin et al. successfully integrated a PEDOT:PSS OECT into a flexible microfluidic system to design a highly sensitive and label-free DNA biosensor [119]. Specifically, the OECT was prepared on a PET substrate, and then, a PDMS microfluidic was attached on the top (Figure 7a). The OECT-microfluidic biosensor showed a high flexibility and stability in which both sides can be very easily bent without reduced charge transport properties (Figure 7b). Furthermore, this OECT device could recognize the DNA targets at low concentrations of 1 nM, and its LOD was highly enhanced (~10 pM) due to integration in the microfluidic channel. In the most recent study, Fu’s group successfully developed an ultrasensitive, portable, and smartphone-controlled RNA biosensor based on an OECT device for early detection of viruses and diseases [120]. This portable sensor was designed with three main components: flexible and transparent miRNA OECT sensor, a meter with readout circuit, and a smartphone (Figure 7c). The PEDOT:PSS OECT sensor was attached into the meter remotely controlled and communicated by the smartphone via Bluetooth. The gate electrode of OECT-based RNA biosensor was modified and functionalized with capture DNA, miRNA, and DNA probe and catalyzed by H2O2 in the aqueous electrolyte (Figure 7 c,d). The devices presented a high sensing performance to miRNA cancer biomarkers in a low volume of sample and low concentration due to its inherent amplification function. These results demonstrated that both OFET and OECT devices have high potential for the development of portable, flexible, ultrasensitive, fast, and low-cost devices in detection viruses.

## 5. Conducting Polymer Hydrogel (CPH)-Based Biosensors

**Figure 8:**
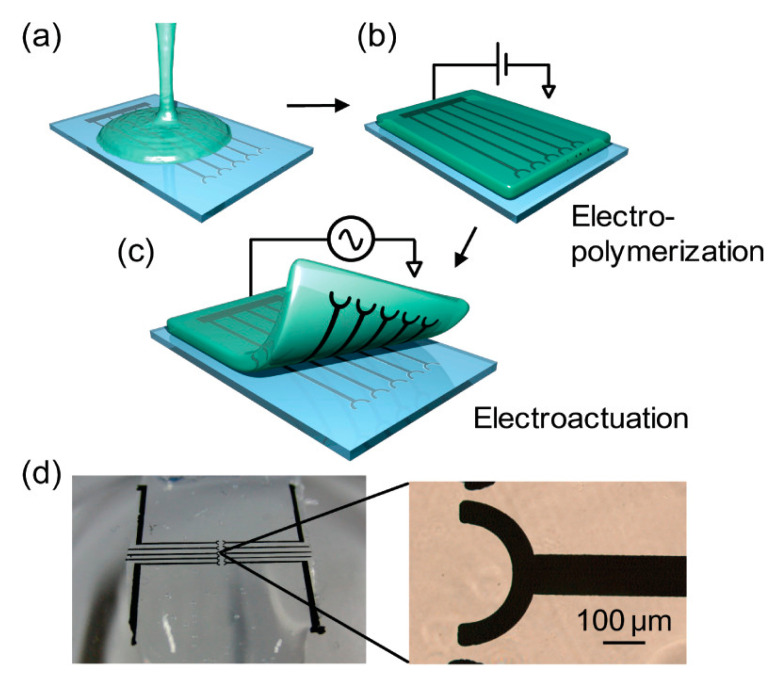
Schematic illustrations of the fabrication of a PEDOT-agarose CPH electrode: (**a**) pouring a melted agarose solution onto a Pt microelectrode substrate; (**b**) electro-polymerization of PEDOT into the gel; and (**c**) electro-actuation several times for peeling. (**d**) Optical image of the PEDOT microelectrode array on the gel sheet [124].

CPHs are considered as advanced materials synergizing advantages of both CPs and 3D hydrogels [121,122], thereby endowing them with great electrical conductivity, mechanical flexibility, high stretchability, biocompatibility, and ease of processing [123]. Among various CPs, PEDOT, PANI, and PPy with advantages of biocompatibility, high capacitance, and flexibility have been represented as attractive electrode materials for the design of electronic implanted devices [124], which have been employed in biomedical applications. For instance, a PEDOT/agarose CPH electrode was successfully fabricated by two electrochemical processes: (i) PEDOT electropolymerization into the agarose hydrogel and (ii) electrochemical actuation-assisted peeling (Figure 8) [124]. The PEDOT/agarose electrode also showed a high stability in water for more than a month. CPHs emerged to be candidates for high performance biosensors owing to facilitating interfaces of electrochemical bio-electrodes. Moreover, CPHs possess hierarchical nanostructures, open porous structure, and large surface area, thereby possibly improving the permeability of bio-substrates and enhancing the interfacial area of electrodes. Therefore, CPH-based biosensors are expected to present highly interesting characteristics such as rapid response, flexibility, stability, high sensitivity and selectivity, and low LOD. Importantly, CPHs can easily pattern any desired shapes at its gelation on various substrates by printing technologies or spraying techniques [125]. Hence, CPHs have been regarded as key functional components for producing scalable bioelectronic sensors and multiplexing biosensor arrays.

Many studies have employed CPHs for the design and development of advanced biosensors in the effective detection of virus antigens. For instance, a sensitive, rapid, and antifouling biosensor for diagnosing clinical miRNA biomarkers directly in biological media was recently fabricated by the assembly of PANI and phytic acid (PA) via one-step electrochemical copolymerization method based on dynamic boronate bonds [126]. PA plays an important role in providing a good biocompatibility and antifouling property for the hydrogel, while PANI enables it with inherent electroactivity, excellent conductivity, and high chemical stability. Thus, the PANI/PA-based CPH exhibited excellent antifouling property and electrochemical characteristics. Importantly, this CPH not only possesses many active sites for attaching DNA but also induces the electrochemical signals by the inherent reduction and oxide signal from PANI. An electrochemical biosensor with both antifouling ability and good selectivity to miRNA was also constructed by the immobilization of DNA bioreceptors onto the PANI/PA hydrogel surface by covalent bond modification. This CPH biosensor worked based on the sensing signals from redox currents of PANI, which is induced by DNA/RNA hybridization reaction. Based on the aforementioned properties of PANI/PA hydrogel, the electrochemical biosensor showed a high sensing performance to microRNA mismatches in a wide liner range of concentration (1.0 fM–1.0 pM) and low LOD (0.34 fM). Furthermore, the promising approach in this study has been proposed to apply for development of clinical diagnostics and biomedical devices in detection of COVID-19 owing to superior advantages of the PANI/PA CPH including good antifouling ability, mechanical flexibility, biocompatibility, high stability and stretchability, facile processability, and high sensitivity and selectivity.

CPHs have been considered as unique smart materials in bioelectronics due to their complete satisfaction with the severe requirements of advanced sensing devices and being suitable for next-generation wearable, implantable, and portable bioelectronics [127,128]. CPHs can show excellent patterning capacity in various substrates due to their high stretchability. For instance, Lu and coworkers patterned a PEDOT:PSS CPH with high conductivity, stability, and stretchability onto a flexible PET substrate by a simple method involving mixing volatile additive dimethyl sulfoxide (DMSO) into aqueous PEDOT:PSS solutions and then controlling dry-annealing and rehydration [129]. In addition, the CPHs also possessed high long-term mechanical and electrochemical stability in wet physiological conditions due to their soft, wet, and conformable properties, thereby imparting highly compatible electrical interfaces between biology and electronics and matching soft tissues and organs [130]. Therefore, CPHs have offered a new opportunity for the development of next-generation advanced bioelectronic devices based on CPs. For example, Arthur et al. reported an OTFT bioelectronic device using a pure PEDOT:PSS CPH as gate electrodes [131]. The study demonstrated that PEDOT:PSS hydrogel gate electrodes showed good transistor characteristics and enabled the transistor device to operate at low voltages, along with excellent sensitivity and selectivity. In summary, CPHs represent a promising material in the development of soft and conformable biosensors.

## 6. Conclusions

Innovative technologies for the development of biosensors with excellent ultra-sensitivity, specificity, portability, and wearability have gained great interest from the community in the management of the rapidly spreading COVID-19 pandemic and others. Many reported studies have demonstrated that biosensors presented a high possibility for early diagnosis, on-site, rapid, and ultrasensitive detection of different virus antigens. CPs have recently started to be commonly employed for the development of advanced biosensors because of their superior advantages involved in their unique properties and high sensing features to virus antigens. There have been numerous CP-based biosensors successfully designed and developed for virus detection, especially for COVID-19. Among them, optical biosensors, i.e., colorimetric and fluorescent sensors, organic thin film transistors (OTFTs), i.e., organic field-effect transistors (OFETs) and organic electrochemical transistors (OECTs), conducting polymer hydrogels (CPH) have demonstrated as the most potential approaches for fabrication of advanced biosensors with high sensitivity, selectivity, flexibility, and processability, which is summarized and presented in Table 3. Furthermore, the recent research also indicated that bioelectronic devices based on CPs can be regarded as one of the trending technologies in the development of novel tools for the early and accurate identification of the virus pandemics. Particularly, flexible, wearable, and stretchable bioelectronic devices integrated CPs may be fabricated and served as a promising technology for virus detection at the POCT systems.

## Figures and Tables

**Table 1:** A comparison of various techniques commonly used for virus detection [7].

Representative Technique	Detecting Target	Advantages	Disadvantages
ELISA	Antibodies or viral antigens	High sensitivity and specificity	Require specific antibodies, multiple steps, and sophisticated laboratories
RT-PCR	Viral genomes	Highest sensitivity, short detection time, best for diagnosis and screening	Expensive, potential sample contamination causing false-positive results, require prior sequence data of the specific target gene of interest
Viral cultivation and isolation	Virions and viral antigens	Gold standard for viral identification, allows further tests for other purposes such as genotype confirmation	Slow and technically demanding, prolonged time to obtain results, requires further identification for a positive sample, not all viruses can multiply in cell culture
CP-based biosensors	Virions, viral antigens, antibodies, viral genomes (potential)	Low cost, short response time, biomimetic structure, dual output signals	Moderate sensitivity and selectivity, moderate stability

**Table 3:** Summary of the state-of-the-art applications of CP-based biosensors in detection of viruses.

Biosensors	Conjugated Polymers	Analytes	Sensitivity	Response Time	LOD	Refs
Fluorescent conjugated polymer biosensors						
DNA fluorescent sensor	Cationic PMNT	ssDNA	10 nM	-	1 nM	[61]
DNA fluorescent sensor	Cationic polythiophene	ssDNA	18 zM	5 min	3 zM	[65]
DNA fluorescent sensor	Cationic polythiophene derivatives	Oligonucleotides	2 × 10^−7^ M	5 min	2 × 10^−7^ M	[70]
Fluorescent sensor	Cationic polythiophene derivative (PT)	*E. coli* *S. aureus* *C. albicans*	1.0 × 10^8^ cfu/mL 1.2 × 10^8^ cfu/mL 5 × 10^7^ cfu/mL	25 min	7.5 × 10^7^ cfu/mL 9 × 10^7^ cfu/mL 4 × 10^7^ cfu/mL	[132]
Label-free DNA sensor	Poly(3-alkoxy-4-methylthiophene)	Hepatitis B virus	100 pmol/L	45 min	88 pmol/L	[133]
Forster energy transfer (FRET) sensor	Water-soluble conjugated polymers	ssDNA	2.1 × 10^−8^ M	-	5.1 × 10^−7^	[134]
Colorimetric conjugated polymer-based biosensors						
Colorimetric RNA biosensor	Polythiophene derivative (PMNT)	MicroRNAs	0.05–1.0 mM	25 min	10 nM	[94]
Colorimetric naked-eye biosensor	Polydiacetylene	H1N1 virus	2.75 × 10^6^–6.8 × 10^5^ PFU	-	10^5^ PFU	[80]
Colorimetric vesicle sensor	Polydiacetylene	Oligonucleotides	2 nM–20 µM	-	2 nM	[83]
Colorimetric vesicle sensor	Polydiacetylene	H5 influenza virus	1.35 copies/µL	20 min	0.53 copies/µL	[86]
Substrate colorimetric biosensor	Polyvinylidene difluoride (PVDF)-supported polydiacetylene	Foot-and-mouth disease virus	7.6–122 μg/mL	10 min	7.6 μg/mL	[89]
Liposome colorimetric biosensor	Polydiacetylene liposome	Antibody of bovine viral diarrhea virus	0.001 to 100 μg/mL	24 h	0.001 μg/mL	[90]
Visible colorimetric biosensor	Polydiacetylene vesicles	Hepatitis B surface antibody	0.1–1 ng/mL	15 min	0.1 ng/ml	[91]
Flow-through colorimetric biosensor	Cationic poly (3-alkoxy-4-methylthiophene)	Lung cancer (microRNA) Hepatitis B virus DNA	1 nM to 10 mM 1 nM to 10 mM	-	~0.6 nM ~2 nM	[96]
Label-free colorimetric biosensor	Polydiacetylene	Influenza antigens	3.3–33 µg/mL	10 min	3.3 µg/mL	[97]
Multiple biosensor chip	Polydiacetylene liposome	Six species of pathogen	10^2^–10^6^ units/mL	30 min	10^2^ units/mL	[99]
Paper colorimetric sensor	Polyaniline	Escherichia coli	8.56 log CFU/mL	80 min	0.52 log CFU/mL	[101]
Paper colorimetric chip	Polydiacetylene	Influenza A (pH1N1) virus	5 × 10^4^ TCID_50_	3 h	5 × 10^3^ TCID_50_	[103]
CP-based organic thin film transistors						
Water-gated OFET	Poly(3-hexylthiophene)	DNA (HIV virus)	1–100 nM	2 h	1 nM	[116]
Ultraflexible OFET	DPh-BBTNDT	C-reactive protein (CRP) antigen	500 ng/mL	100 s	1 μg/mL	[117]
OECT sensor	PEDOT:PSS	Human influenza virus	0.025–1 HAU	30 min	0.025 HAU	[118]
Flexible Microfluidic OECT	PEDOT:PSS	ssDNA	1 nM	6 h	10 pM	[119]
Portable OECT sensor	PEDOT:PSS	microRNA	10^−6^–10^−14^ M,	1 h	10^−14^ M	[120]
Conducting polymer hydrogel (CPH)- based biosensors						
CPH sensor	PANI	microRNA	1.0 fM–1.0 pM	45 min	0.34 fM	[126]

## Data Availability

Not applicable.
